# WO_3_-Based Thin Films Grown by Pulsed Laser Deposition as Gas Sensors for NO_2_ Detection

**DOI:** 10.3390/s24227366

**Published:** 2024-11-19

**Authors:** Alessandro Bellucci, Angela De Bonis, Mariangela Curcio, Antonio Santagata, Maria Lucia Pace, Eleonora Bolli, Matteo Mastellone, Riccardo Polini, Raffaella Salerno, Veronica Valentini, Daniele M. Trucchi

**Affiliations:** 1 DiaTHEMA Lab, CNR-ISM, Montelibretti Branch, Via Salaria km 29.300, Monterotondo, 00015 Rome, Italy; eleonora.bolli@ism.cnr.it (E.B.); raffaella.salerno@ism.cnr.it (R.S.); veronica.valenti@ism.cnr.it (V.V.); daniele.trucchi@ism.cnr.it (D.M.T.); 2 Dipartimento di Scienze, Università degli Studi della Basilicata, Via dell’Ateneo Lucano 10, 85100 Potenza, Italy; angela.debonis@unibas.it (A.D.B.); mariangela.curcio@unibas.it (M.C.); 3 FemtoLAB, CNR-ISM, Tito Scalo Branch, Zona Industriale, Tito, 85050 Potenza, Italy; antonio.santagata@ism.cnr.it (A.S.); marialucia.pace@ism.cnr.it (M.L.P.); matteo.mastellone@ism.cnr.it (M.M.); 4 Dipartimento di Scienze e Tecnologie Chimiche, Università di Roma ‘Tor Vergata’, 00133 Rome, Italy; polini@uniroma2.it

**Keywords:** pulsed laser deposition, thin films, tungsten oxide, gas sensing, surface oxygen vacancies, thermal annealing

## Abstract

Thin films based on tungsten oxide (WO_3_) were grown by nanosecond pulsed laser deposition on alumina printed-circuit boards to fabricate electrochemical sensors for nitrogen dioxide (NO_2_) detection. Samples exposed to thermal annealing (400 °C for 3 h) were also produced to compare the main properties and the sensor performance. Before gas testing, the morphology and structural properties were investigated. Scanning electron microscopy and atomic force microscopy showed the formation of granular films with a more compact structure before the thermal treatment. Features of the main WO_3_ phases were identified for both as-deposited and annealed samples by Raman spectroscopy, whereas X-ray diffraction evidenced the amorphous nature of the as-deposited samples and the formation of crystalline phases after thermal annealing. The as-deposited samples showed a higher W/O ratio, as displayed by energy-dispersive X-ray spectroscopy. An Arrhenius plot revealed a lower activation energy (0.11 eV) for the as-deposited thin films, which are the most electrically conductive samples, presenting a better gas response (30% higher than the response of the annealed ones) in the investigated NO_2_ concentration range of 5–20 ppm at the moderate device operating temperature of 75 °C. This behavior is explained by a larger quantity of oxygen vacancies, which enhances the sensing mechanism.

## 1. Introduction

The fabrication of sensors based on nanostructured resistive-type metal oxide semiconductors is becoming one of the most studied solutions for gas detection, due to their simple sensing principle, i.e., a variation in the surface electrical resistance due to the adsorption of gases, together with the capability of achieving a high surface-to-volume ratio for the active material [1,2]. Tungsten oxide (WO_3_), an n-type metal oxide semiconductor (bandgap of 2.6–2.8 eV), represents one of the most investigated materials used to act as a thin-film gas sensor for different gas analytes, such as NO_x_ [3,4], NH_3_ [5], H_2_S [6], H_2_ [7], CO [8], and more [9,10]. The properties and the response of WO_3_-based gas sensors strongly depend on the method of synthesis, which influences grain size, porosity, and electrical characteristics. Various and complex morphologies of WO_3_ are reported in the literature, such as nanorods [11], nanosheets [12], or nanoplates [13]. Additionally, different approaches for improving gas sensitivity and selectivity have been carried out, such as doping, surface functionalization, or the formation of heterojunctions with low-dimensional materials [14]. Among the deposition methods, sputtering [15], thermal evaporation [16], sol–gel [17], chemical vapor deposition [18], and electrodeposition [19] are the most diffused for the growth of WO_3_ thin films. However, despite several promising results for fabricated WO_3_ thin-film sensors [20,21], most of the reported types of gas sensors operate at medium-high temperatures (≥100 °C), thus complicating the device architecture for operations in relevant conditions for long periods. Instead, lower operating temperatures for the device are beneficial for fabricating low-power-consumption sensors, as well as improving the compatibility with the electronic chain for compact detection systems.

In this work, we prepared WO_3_ thin films (with and without thermal annealing) grown on alumina printed-circuit boards (PCBs) by means of nanosecond pulsed laser deposition (ns-PLD) to be tested as gas sensors for nitrogen dioxide (NO_2_), which is one of the most relevant gases to be detected for pollution in industrial and automotive applications, like the release from battery instability behavior [22], and potentially harmful for human health [23]. The PLD technique has gained significant importance in developing a diverse range of innovative materials, including superconductors, semiconductors, ceramics, and alloys, characterized by well-defined compositions, phases, and properties [24]. This technique allows the properties of the coatings to be controlled by changing deposition parameters such as laser fluence, substrate temperature, gas pressure, or distance between target and substrate. Both nanosecond (ns) and femtosecond (fs) pulses exhibit versatility in providing materials suitable for numerous applications, such as catalysis, energy conversion or storage, and fabrication of plasmonic, electronic, thermionic, thermoelectric or photonic devices [25,26,27]. These materials can be synthesized under different environmental conditions, including vacuum, inert, or reactive background gases, as well as in liquid environments. Although in many studies the growth of WO_3_ coatings occurs under an oxygen-controlled atmosphere, in this work the deposition was conducted in vacuum at a pressure of 10^−4^ Pa.

Even if the absence of reactive gas during the process simplifies the experimental conditions, the properties of the produced material must be studied to understand if the desired characteristics can be achieved. In this study, thermal annealing has also been performed to analyze the effect on the performance of thin films. After the analysis of the physico-chemical properties of the thin films fabricated by ns-PLD, the NO_2_ response of the WO_3_-based gas sensors is herein reported to demonstrate the devices’ operation at moderately low temperatures (≤75 °C) with a significant value of sensitivity.

## 2. Materials and Methods

### 2.1. Preparation of WO_3_ Thin Films

The second harmonic (532 nm) of the Nd-YAG laser source (Handy-YAG; Quanta System, Milano, Italy) was used for deposition experiments. The laser, operating with a repetition rate of 10 Hz, a pulse duration of 7 ns, and a fluence of 20 J/cm^2^, was directed into a stainless-steel vacuum chamber and focused on the target substrate obtained by cold-pressing WO_3_ powder (Merck KGaA, Darmstadt, Germany) through a 350 mm-focus lens. The chamber was evacuated by a scroll–turbomolecular vacuum system to a background vacuum of 10^−4^ Pa. The substrates were placed 3 cm away from the target, and the depositions lasted for 2 h with the substrate kept at room temperature, leading to a nominal film thickness of 300 nm. A post-deposition thermal annealing at 400 °C for 3 h in air was conducted on selected samples.

### 2.2. Characterization of the Physico-Chemical Properties

X-ray diffraction measurements were performed by means of a D5000 Siemens (Munich, Germany) instrument, using a Cu Kα1 radiation source (λ = 1.5405600 Å). The following conditions were applied for the investigation: 2θ = 20–60°, step size 0.040°, time per step 4 s.

Raman measurements were performed using a Horiba Scientific Ltd (Kyoto, Japan) LabRam HR Evolution confocal spectrometer equipped with a 100 mW Oxxius (λ_exc_ = 532 nm) laser source, a computerized XY table, and an electron-multiplier CCD detector. A grating with 1800 grooves/mm and an Olympus U5RE2 microscope with a 100× objective (numerical aperture of 0.9) were used. All Raman spectra were recorded in backscattering geometry focalizing 10% of the laser power (10 mW) on the sample (laser spot on the sample surface 0.7 μm). Twenty spectra with an accumulation time of 10 s were averaged for the measurements performed on the sensors measured before and after gas testing.

Field-Emission Gun Scanning Electron Microscopy (FEG-SEM, Zeiss, Oberkochen, Germany, LEO Supra 35 and ThermoFisher, Waltham, MA, USA, SCIOS2 FIB-SEM apparatus) and Atomic Force Microscopy (AFM, OmegaScope platform, Horiba Scientific, Irvine, CA, USA) were used to study the morphology of the WO_3_ thin films. AFM imaging was carried out in tapping mode using a silicon pyramidal tip (MikroMasch, Sofia, Bulgaria, HQ:NSC15/Al BS) with a characteristic radius of ~8 nm. The resonance frequency was 325 kHz, whereas the operational amplitude was set at 60 nm. All the AFM data were acquired by fixing the scan rate at 1 Hz, then filtered and analyzed using the AIST-NT SPM v3.5.160 control software.

EDS measurements were carried out using the ThermoFisher Scientific UltraDry 129 eV 60 M detector, which has a crystal active area of 60 mm^2^, mounted on the ThermoFisher SCIOS2 FIB-SEM apparatus. A fixed acceleration voltage of 10 kV was applied to a 17.41 µm^2^ examined image area, and the Pathfinder X-ray Microanalysis 2.11 software was employed.

### 2.3. Electrical and Gas Sensing Measurements

**Figure 1:**
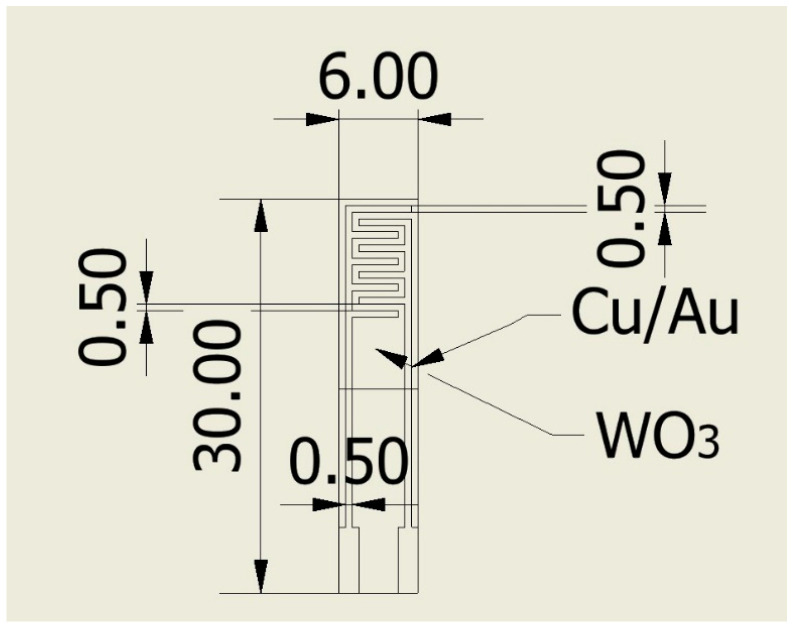
Design for the WO_3_-based NO_2_ sensors on an alumina PCB. The WO_3_ thin film is deposited on the metallic fingers (i.e., half of the PCB) by using a mechanical mask. The values shown in the sketch are reported in mm.

An alumina (thickness: 1 mm) printed-circuit board (PCB) with two copper/gold electrodes was used as substrate for the deposited WO_3_, forming the device for the gas sensing measurements, according to the design shown in Figure 1. The inter-electrode gap at the minimum point between the two electrodes is 0.5 mm. On the rear of the PCB, graphite electrodes were provided for heating the sensor (about 5 W consumed for reaching the maximum temperature of 200 °C). A convenient push-and-pull connection of the electrical contacts to the signal conditioner was obtained by soldering the connector directly to these contacts.

**Figure 2:**
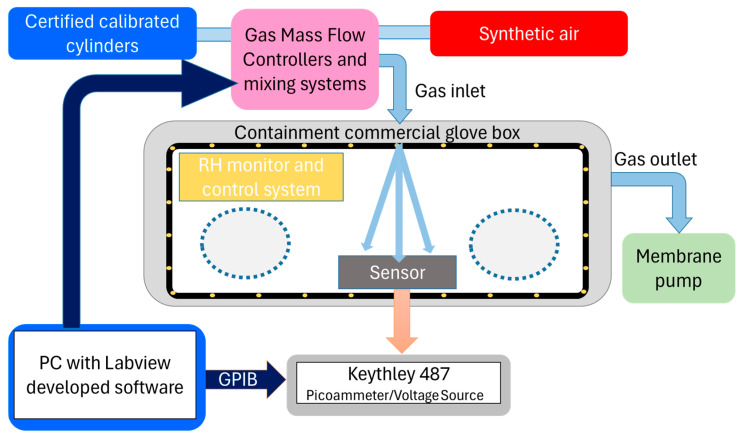
Sketch of the experimental setup. The system is composed of a commercial glove box equipped with an RH monitor and control system. For the gas exposure and the chamber evacuation, two lines are connected, one to a gas mixing system and the other one to a membrane pump. The gas concentration is obtained by mixing the flows from a synthetic air cylinder and calibrated cylinders (certified by the company Nippon Gases, Anagni, Italy, which provided the gas mixture) that are regulated by mass flow controllers. The sensor is electrically connected to an electrometer (Keithley 487), and the data acquisition is performed via a PC with customized software able to control and register the gas exposure, the electrical signals, and the RH level.

A commercial glove box (Cole-Parmer Instrument Company, Vernon Hills, IL, USA) with a volume of about 0.25 m^3^ was used for the gas sensor tests. The sketch of the experimental setup is shown in Figure 2 and also described elsewhere [28]. Thanks to the use of calibrated flowmeters (one on the gas line for the NO_2_ gas cylinder already mixed at 20 ppm and one for that of the synthetic air gas cylinder) and a precise controller, it was possible to regulate the flow of NO_2_ according to the 5–20 ppm concentration range in the chamber, which was evacuated by a membrane pump (pressure of ~10 Pa) to assure both stable conditions and fast removal of the gas during the different cycles of measurement. The relative humidity was monitored and controlled to be in the range of 50 ± 5% RH by a dedicated automatic humidity control system (humidity sensor, humidifier, and desiccant drying system). Such RH range was selected because these are typical values for indoor conditions. An electrometer (Keithley, Tektronix, Beaverton, OR, USA, 487 Picoammeter/Voltage Source) was used for measuring the electrical resistance. To control the measurements, customized software specifically developed with the LabVIEW (National Instruments, Austin, TX, USA) language was used to record data in an automated way. The same electronic measurement chain was used for the Arrhenius analysis of the resistance evaluation as a function of the sensor temperature.

## 3. Results

**Figure 3:**
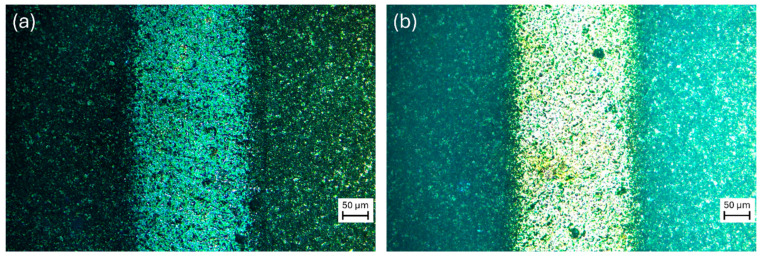
Optical images of (**a**) as-deposited and (**b**) annealed films acquired around a finger (Au) of the PCB by using a 20× objective coupled to a DM6 M LEICA optical microscope and the LEICA EL6000 UV-Vis fluorescent light source.

A visual inspection provided initial information about the effect of thermal annealing by comparing the different samples. The as-deposited films appear dark (towards a dark gray tone), whereas after the annealing, the films look transparent (i.e., white). Figure 3 shows optical images under UV-Vis fluorescent light source, showing the different optical properties of the two films qualitatively.

**Figure 4:**
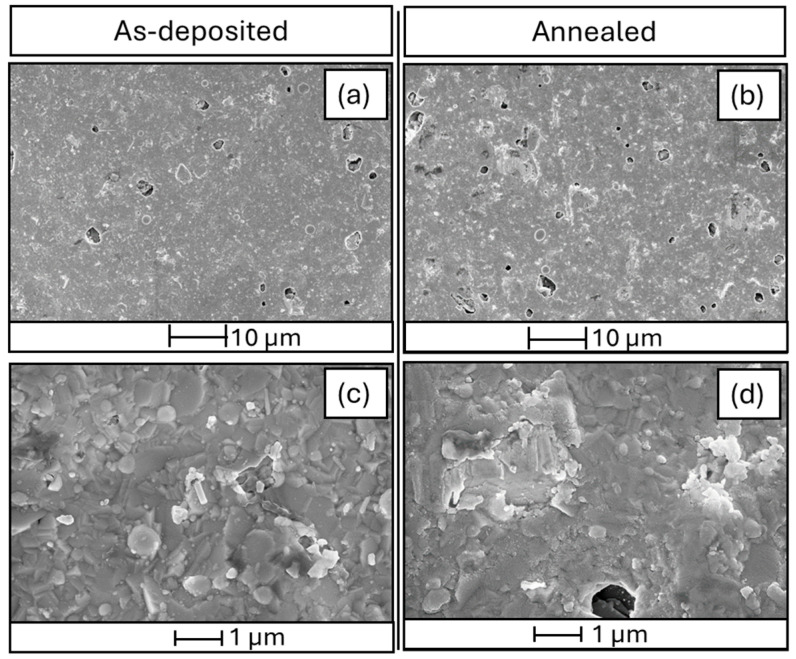
SEM images of (**a**,**c**) as-deposited films and (**b**,**d**) annealed thin films.

Figure 4 shows the morphologies of the thin films investigated by FEG SEM. The micrographs evidence the formation of granular films, with an average feature nanometric grain size of 100 ± 30 nm (calculated using the freely available software for image analysis and processing ImageJ v1.54i) and the presence of some micron-sized droplets or agglomerates and pores (i.e., lack of materials). The as-deposited samples result in a more compact structure (i.e., with a lower average number of pores), even if a predominant shape for the nanostructures cannot be distinguished in both cases. However, the random spatial orientation at the nanoscale should provide enhanced surface reactivity and fast gas diffusion, which are required for good gas sensing performance.

**Figure 5:**
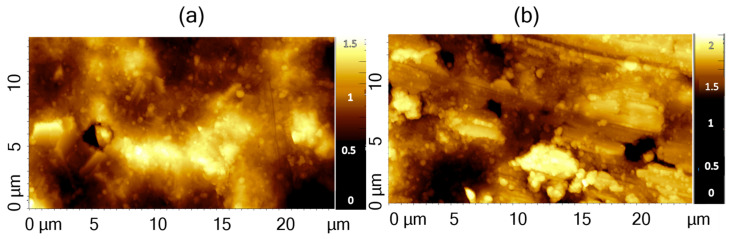
AFM topographies of as-deposited (**a**) and annealed (**b**) WO_3_ samples deposited on Al_2_O_3_.

Additionally, AFM was used to investigate and quantify the samples’ roughness, which is one of the most informative parameters for gas sensors. The comparison of the different thin films does not allow identifying features specific to each sample (Figure 5), which presents similar values of roughness (146 nm and 132 nm for the as-deposited and annealed samples, respectively) and a ratio of the effective surface area to the investigated geometrical area equal to 1.1 for both samples. This means that the nanostructuring increases the available surface for the detection of 10% with respect to a possible flat film. This similar behavior is interesting since the annealing treatment is expected to modify the grain size [29], whereas in this case, it seems to keep the overall surface structure unaltered.

**Figure 6:**
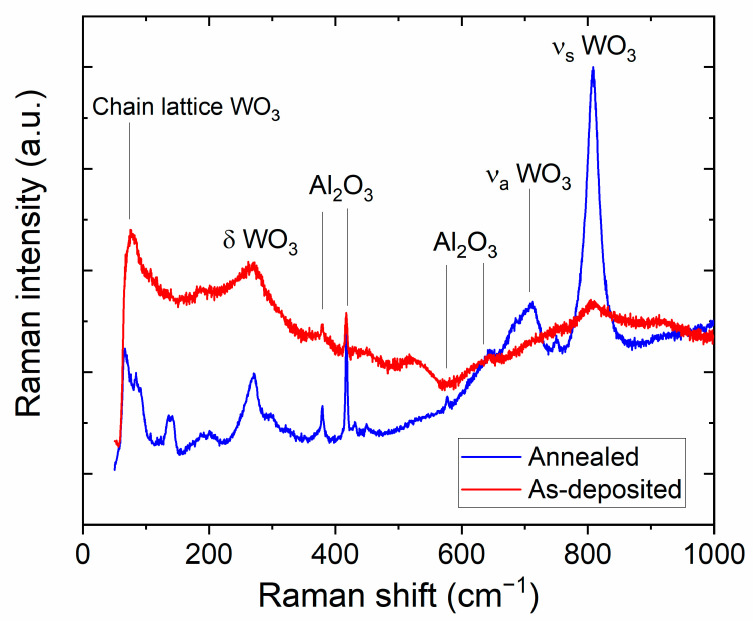
Raman spectra of as-deposited and annealed WO_3_-based thin films on alumina substrates.

The Raman spectra of the films are shown in Figure 6, where the WO_3_ main bands at 75, 271, 711, and 807.5 cm^−1^ are detected for both samples. The band at 807.5 cm^−1^ is assigned to the symmetric stretching mode of the WO_3_ monoclinic structure [30], whereas the band at 711 cm^−1^ arises from the asymmetric stretching vibrations of WO_3_ [31]. The band at 271 cm^−1^ is attributed to the O-W-O bending mode of vibrations [32]. Moreover, the band at 75 cm^−1^ may be assigned to the (W_2_O_2_)_n_ chain into the lattice of WO_3_ [33].

The Raman analysis indicates a better crystallization of WO_3_ for the annealed sample, with the identification of sharper peaks (lower values of full width at half maximum for most of the peaks). Conversely, the main bands for the as-deposited films are significantly reduced in intensity and certainly broadened. This is possible to explain by considering that the as-deposited films show coalescence of the formed nanostructures, resulting in an increased light absorption and a consequential decrease in the Raman signal collected by the detector. Interestingly, the thermal annealing performed in air does not induce further oxidative phenomena (no different bands appear that could be attributed to differently oxidized phases compared to the initial WO_3_).

**Figure 7:**
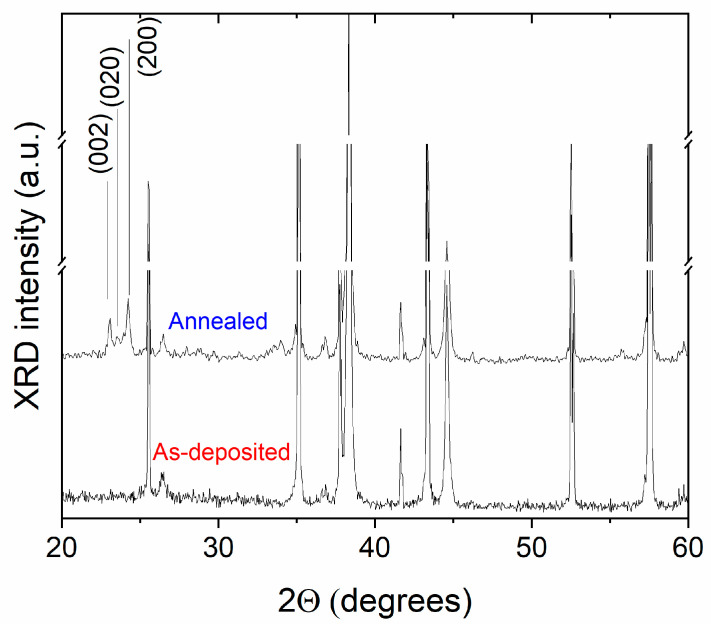
XRD spectra of the as-deposited and annealed WO_3_-based thin films on alumina substrates in the range 2θ 20–60°. The peaks not labeled by the crystallographic orientation correspond to the Al_2_O_3_ substrate.

To confirm this hypothesis, XRD measurements were performed (Figure 7). The crystalline phase was detected only for the annealed sample, whereas the as-deposited film presents an amorphous nature (in the spectrum, the only identified peaks, which are not labeled in the figure, correspond to the Al_2_O_3_ substrate). From the XRD pattern, the peaks in the 2θ range 20–25° are the reflections of the (002), (020), and (200) crystal planes of monoclinic WO_3_ (JCPD 01-089-4476), which is known to be the most stable phase for the WO_3_ system.

**Figure 8:**
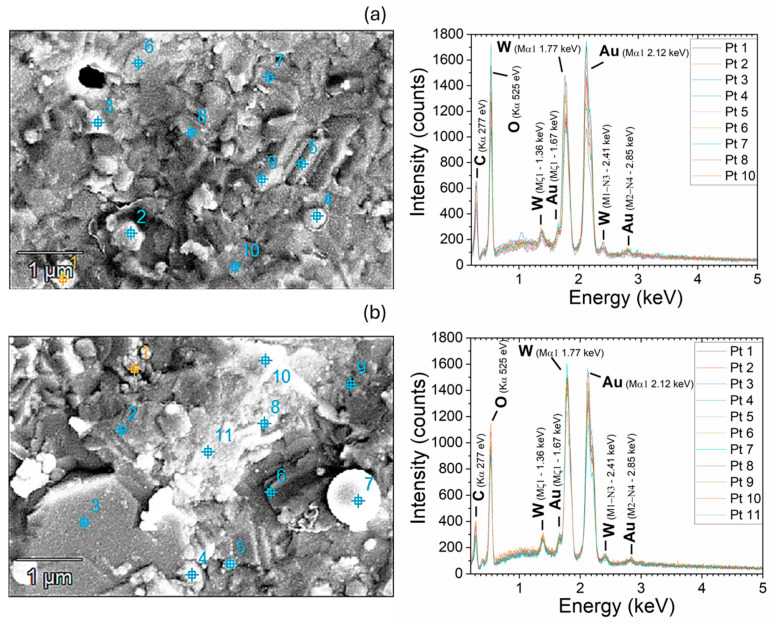
SEM maps and EDX spectra (based on the points highlighted in the SEM image) for the annealed (**a**) and as-deposited (**b**) samples.

EDX measurements were performed on the samples to verify the chemical composition of the structures. Since the morphology presents particles of different sizes, a statistical analysis was carried out by acquiring spectra on several positions on the films deposited on the Au-coated electrodes. The average values of the detected chemical elements are reported in Table 1, whereas an exemplified map with the EDX spectra is shown in Figure 8.

From this analysis, it appears that the quantity of the ratio W/O is generally higher in the as-deposited films than in the annealed ones. It is important to state that no signal from Al was detected, thus indicating that the volume investigated by EDX is stopped in the Au electrodes without contribution of the alumina substrate. Even if a quantitative analysis cannot be carried out (the %Au is higher in the annealed sample with respect to the as-deposited one, meaning that probably the measured volume is not the same), the oxygen deficiency for the as-deposited sample is clearly distinguished. Correlating the results obtained from Raman spectroscopy (which identified the presence of WO_3_ on both samples), XRD (which shows crystalline phases only after thermal annealing), and EDX, it is probable to infer that the ns-PLD technique induces the formation of amorphous and non-stoichiometric structures, like WO_3−x_, for the deposited films. Indeed, the different stoichiometry is reported for WO_3_ systems with different colors [33], as it is the case of the samples in this work (from the dark as-deposited films to the transparent and crystalline films after thermal annealing).

**Figure 9:**
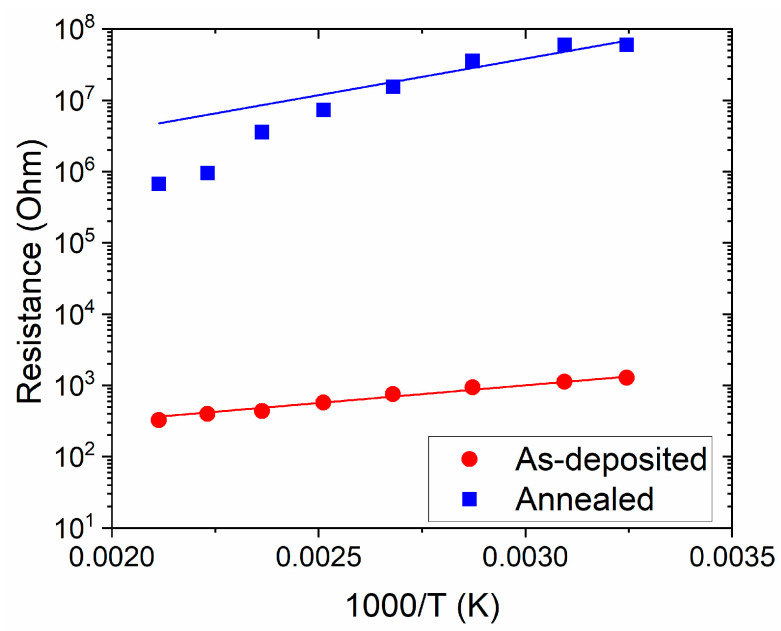
Arrhenius plot of WO_3_-based thin films’ electrical resistance in the temperature range RT–200 °C.

Before gas testing, electrical resistance (R_0_) measurements were performed in vacuum (~0.1 Pa) as a function of temperature (T) from room temperature (RT) to 200 °C, with the aim of determining the activation energy (E_A_) using the Arrhenius formula (Equation (1)):

R_0_ = R’∙exp(E_A_/(k_B_∙T)), (1)

where R’ is the pre-exponential factor and k_B_ is the Boltzmann constant. For both samples, the resistance decreases when the temperature increases, as expected for a semiconductor. The as-deposited sample is more electrically conductive than the annealed one, with an activation energy of 0.11 eV calculated from the application of a best fit (Figure 9). This value is lower than that of the annealed film (0.36 eV).

From an electrical point of view, the sub-stoichiometry of the as-deposited films is demonstrated to be beneficial for the transport of the charge carriers. Considering the results obtained from the physico-chemical analyses, we can conclude that the electrical data are indicative of a large presence of surface oxygen vacancies (V_O_s), as displayed for other WO_3_-based complex structures [34], where the formation of V_O_s highly favors the adsorption phenomena on the basis of the NO_2_ sensing mechanisms. A large concentration of this kind of defect is reported for WO_3−x_ structures [35] and also for WO_3_-based systems affected by degradation in crystallinity [36], which are conditions fully compatible with the as-deposited films in this work. The explanation of the recorded enhancement can be found in the oxygen-deficiency conditions induced by the ns-PLD technique conducted under vacuum without an oxygen flux, which natively promotes a boost in the presence of V_O_s.

**Figure 10:**
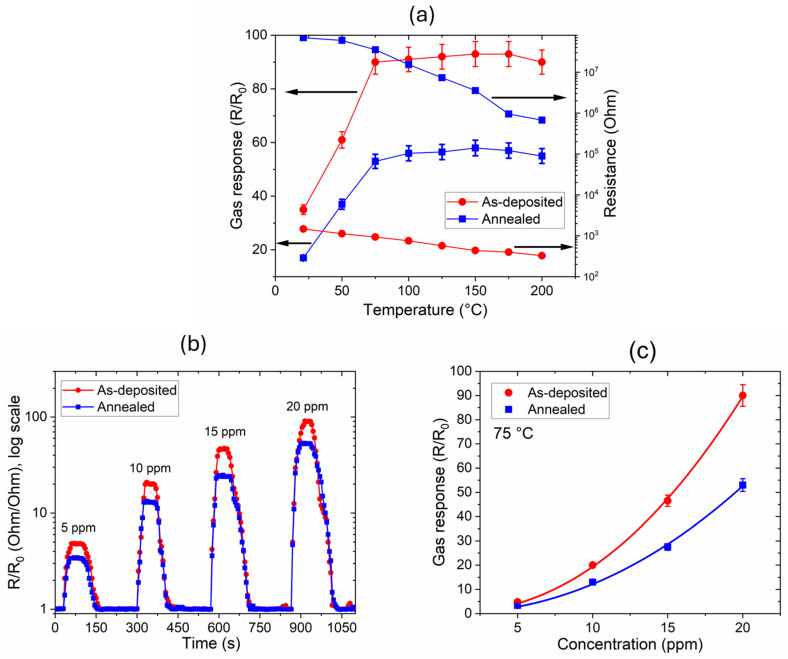
(**a**) Gas response curves (left arrows) and variation in resistance R_0_ (right arrows) for the WO_3_ thin-film sensors as a function of the operating temperatures for 20 ppm concentration of NO_2_; (**b**) dynamic response curves for the gas sensors and different gas concentration values at the fixed temperature of 75 °C; (**c**) gas response curves as a function of concentration at the fixed temperature of 75 °C considering the saturation point taken from the dynamic curves.

The sensor gas response (defined as R/R_0_, where R is the sensor resistance upon gas exposure compared to the baseline resistance R_0_) was studied in the concentration range 5–20 ppm and by varying the sensor temperature. First, the gas response was analyzed as a function of temperature, as shown in Figure 10a. As expected from the well-known gas sensing mechanism for n-type metal oxide semiconductors in an oxidizing environment (like nitrogen oxide compounds, NO_x_), the electrical resistance increases when the material is exposed to the gas [20]. The value of R/R_0_ increases up to 150 °C, where the maximum values of 93 and 58 are reached for the as-deposited and annealed samples, respectively. However, contrary to other proposed systems [13,37], the increase is below 5% in the range 75–150 °C, so the following characterization of the sensors will be performed at the fixed temperature of 75 °C, with the aim of obtaining suitable performance while keeping the operating temperature below 100 °C.

Figure 10b shows the dynamic response of the thin-film sensors at different NO_2_ concentrations ranging from 5 to 20 ppm. The gas response increases with an increase in NO_2_ concentration, with a similar behavior shown for both samples. As it can be observed in Figure 10c, a power fit (*y *=* a *×* C^b^*, where *C* is the gas concentration and *a*, *b* are the fitting parameters) is applied, similarly to the interpolation from a Freundlich isotherm (*a* = 0.09 ± 0.03 and 0.12 ± 0.03 for the annealed and as-deposited sensors, respectively). A quadratic dependence (*b* = 2.11 ± 0.10 and 2.21 ± 0.15 for the annealed and as-deposited sensors, respectively) is calculated, thus indicating that the proposed sensors are progressively more sensitive at high NO_2_ concentrations. Regarding reversibility, the response transient curve of the sensors shows that the resistances recover to the initial values after the removal of NO_2_, with an average recovery time, calculated as the time of reaching at least 90% of the initial value, of 80 ± 10 s for both sensors. Additionally, the sensors has a very fast response, reaching the saturation value at a fixed gas concentration with an average time of 20 ± 5 s, which is in line with the results of the fastest sensors reported in the literature [20,21].

Furthermore, considering the approach proposed in the literature for calculating the detection limit (DT) of the sensors [38,39]—which can be obtained from the formula DT = 3 × [5 ppm]/(R − R_0_)/σ, where σ is the electronic noise obtained during the electrical measurement—a baseline gas concentration of 5 ppm over 10 min of exposure has been investigated. From the resulting signal-to-noise ratio, a DT of 4 ppb has been calculated. This analysis allows us to consider these sensors promising, identifying their target application as the monitoring of NO_2_ in indoor environments such as buildings, where the gas concentration is typically lower than 3 ppm, or industrial plants, where DT values as low as possible are required.

**Figure 11:**
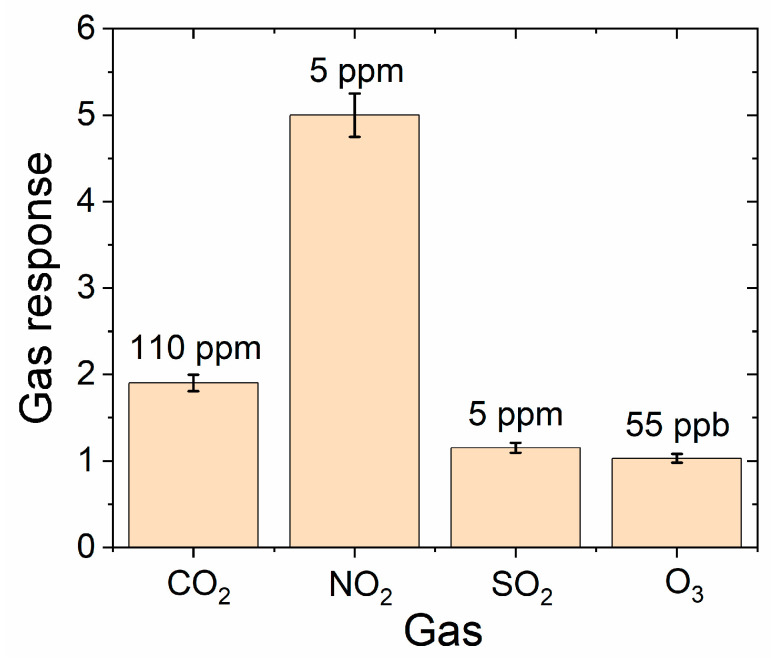
Comparison of gas responses of the as-deposited WO_3_ thin-film sensor to different gases at 75 °C at fixed gas concentration (110 ppm for CO_2_, 5 ppm for NO_2_ and SO_2_, 55 ppb for O_3_). The gas concentration for each gas is the minimum level achievable in the calibrated experimental setup used for this study (labelled on each column for every gas).

In this context, a selectivity study has been carried out to understand the capability of the as-deposited NO_2_ sensors compared to the detection of other gases. Figure 11 shows the results obtained using the proposed system to measure other possible toxic gases present in indoor conditions, like CO_2_, SO_2_, and O_3_. Even if a direct comparison cannot be made because the concentration ranges are different for the gases according to the limitations of the experimental setup in terms of accuracy under the minimum measurable level at which the measurement was performed for each gas, it is possible to note that the WO_3−x_-based sensors are extremely more sensitive to NO_2_, where the response is about five times higher with respect to the other gases. Regarding ozone, whose detection was performed in the ppb range, the gas response is very low (1.05 ± 0.05), and it is difficult to state if the sensor is able to detect it.

**Figure 12:**
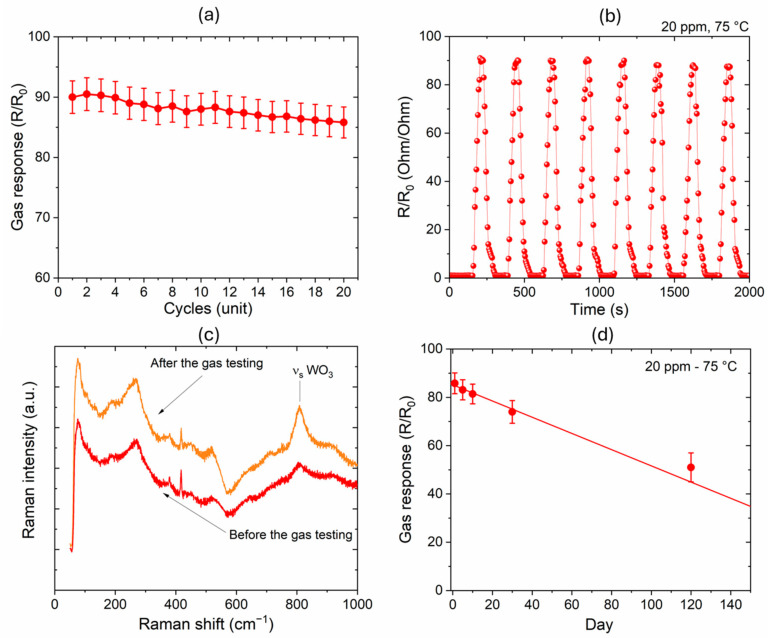
(**a**) Gas response of the as-deposited sample measured at 20 ppm after twenty continuous cycles of gas in/out; (**b**) example of dynamic response curves (eight curves) for the gas sensors at a fixed gas concentration of 20 ppm and a fixed temperature of 75 °C; (**c**) Raman spectra before and after the gas testing; (**d**) gas response of the as-deposited sample measured at 20 ppm on different days of measurements spanned for 4 months.

The stability of the sensors based on the as-deposited thin films was tested by measuring the sensitivity after several continuous cycles of exposure at the maximum gas concentration of 20 ppm (Figure 12a). The gas response slightly decreases by increasing the number of cycles, but the deviation from the starting value is less than 5% (85.8) after the 20th cycle of measurement, as shown in Figure 12b. This indicates a very good stability of the sensor under critical conditions, which is also highlighted by the Raman spectrum of the WO_3−x_ thin films acquired after the test (Figure 12c). In fact, apart from a moderate increase in intensity of the band at 807.5 cm^−1^, which could indicate a modest modification of the structure towards crystallization during the long operating conditions at a temperature of 75 °C, the spectrum remains unaltered after gas testing.

Additionally, to evaluate the lifespan and the reproducibility of the as-deposited sensors, the results of the stability control over the long term and the batch-to-batch reproducibility are reported. Figure 12d shows the gas response at 20 ppm after different days of measurement. A decrease in performance was recorded, quantified in a reduced gas response of 24% (gas response of 69) after four months from the first measurements. It is important to note that the tests performed on days 1, 3, and 5 are prolonged tests in critical conditions (20 cycles at 20 ppm) that could accelerate the deterioration of the surface. However, by applying a fit to the recorded data, we can estimate the maximum time for achieving a gas response higher than 1.2 (considered as the minimum reliable threshold of detection) to be about 254 days.

Finally, three different samples were prepared and compared to evaluate the sensors’ reproducibility. Table 2 shows the results obtained for the different sensors (as-deposited) for R_0_ and R measured under 20 ppm of NO_2_ concentration. This test indicates good batch-to-batch reproducibility, with the recorded differences ranging within an experimental error ≤ 2%.

## 4. Discussion

To evaluate the results obtained in this work, a comparison with the main studies on similar systems (WO_3_ for NO_2_ detection) was made. It is important to note that all the mentioned works report that the change in resistance is an increase in the value when the active material is exposed to NO_2_, which is typical for n-type semiconductors, as already described previously. Therefore, the gas sensing mechanism is related to the adsorption and desorption of gas molecules on the sensor surface, where the electronic concentration varies according to the type of material (p- or n-type) and gas environment (reducing or oxidizing atmosphere) [40]. This well-known working mechanism is based on the following scheme: (1) oxygen molecules are chemisorbed on the surface, reacting with electrons of the WO_3_-based layer and forming active oxygen species (like O^2−^, O^−^, etc.); (2) when NO_2_ gas is present, oxygen species react with the gas by reducing the carrier concentration in the surface material and thus increasing the electrical resistance. The efficacy of this process depends on different factors, such as the presence of oxygen vacancies, electronic defects, adsorption sites, etc. Table 3 shows the most significant solutions developed with different techniques. For sake of clarity, only the works that considered the same method for evaluating the gas response (as R/R_0_) have been presented. As it is possible to observe from the table, most of the deposition methods are chemical synthesis for the formation of 1D and 2D nanoarchitectures. Generally, very significant results were obtained for WO_3_ nanosheets that achieved high values of gas response at very low concentration levels (40 ppb). The use of PLD has the advantage of being a physical method for rapid and scalable depositions for the fabrication of large-area and serial sensors. Additionally, one of the advantages of this work is the reduction in the device’s operating temperature under 100 °C. The objective is to further decrease the temperature down to room temperature, as already reported for different works present in Table 3. One possibility to obtain this condition is to apply doping to the WO_3−x_ matrix, which could enhance the performance, as shown in Ref. [41], or to consider a multilayer approach for the formation of heterostructures with other metal oxide semiconductors (like ZnO [42]), which significantly improves the performance through the engineering of the heterojunction interface. In this context, PLD is a potential technique that can be potentially beneficial for fabricating such different structures.

Finally, the investigation at gas concentrations lower than 5 ppm will be needed in the future to compare the performance with other solutions proposed in the literature and confirm the calculated DT of 4 ppb obtained by applying a statistical method.

## 5. Conclusions

NO_2_ sensors based on WO_3_ thin films were successfully fabricated by the nanosecond pulsed laser deposition technique. Structural and morphological analyses show the formation of a granular amorphous material, which does not present additional chemical phases not belonging to the WO_3_ composition. Thermal annealing (400 °C, 3 h, atmospheric pressure) induces the organization of a crystalline structure in the film. The as-deposited samples result to be more electrically conductive with respect to the annealed ones, probably due to a higher value of the ratio W/O with respect to the nominal stoichiometry, as detected by EDX, which forms non-stoichiometric structures with a high number of electronically active defects, i.e., oxygen vacancies into the WO_3_ film. The sensitivity of the as-deposited sample is 1.8 times higher than that of annealed films at the device’s operating temperature of 75 °C for a gas concentration of 20 ppm. The key factor enhancing sensing performance is attributed to the higher amount of oxygen vacancies, which improve the surface electrical properties of the thin films by increasing the number of charge carriers available for the sensing mechanism. The stability of the fabricated sensors after several cycles of measurement at the maximum concentration of 20 ppm, together with the possibility of keeping the operating temperature at 75 °C, makes these gas sensors appealing for their use in indoor monitoring applications for industrial plants.

## Figures and Tables

**Table 1:** EDX elemental composition (in % atomic weight) of the as-deposited and annealed samples taken on the Au fingers of the sensors.

Sample	%C	%O	%Al	%W	%Au
Annealed	13.69	53.95	-	10.72	21.10
As-deposited	13.49	52.65	-	14.76	18.97

**Table 2:** Values of resistance for three different as-deposited sensors in terms of baseline resistance and resistance under exposure (5 ppm) at an operating temperature of 75 °C.

As-Deposited Sample	Sample #1 (Ω)	Sample #2 (Ω)	Sample #3 (Ω)
R_0_	936.8 ± 4.9	944.4 ± 4.7	941.7 ± 4.7
R (5 ppm)	4680 ± 12.4	4748 ± 18.2	4686 ± 11.3

**Table 3:** Performance of various structures based on WO_3_ in the sensing of NO_2_ gas.

System	Deposition Method	Gas Response	Detected Concentration	Response/Recovery Times	Operating Temperature	References
WO_3_ nanoplates	Hydrothermal	10	5 ppm	-	100 °C	[13]
WO_3_ nanoplates	Hydrothermal	130	100 ppm	-	100 °C	[13]
WO_3_ nanoparticles	Hydrothermal	251.7	5 ppm	11 s/124 s	100 °C	[43]
WO_3_ nanorods	Thermal oxidation	2.02	10 ppm	-	250 °C	[44]
WO_3_ nanosheet	Hydrothermal	30	40 ppb	-	150 °C	[45]
WO_3_ nanorods	DC magnetron sputtering	27	2 ppm	-	250 °C	[46]
WO_3_–ZnO heterostructure	Hydrothermal	186	200 ppm	-	200 °C	[42]
Polyaniline–WO_3_ thin film	Hydrothermal	14.47	20 ppm	-	50 °C	[47]
2 wt % Sb-doped WO_3_	Chemical method	51	10 ppm	-	20 °C	[41]
WO_3_ nanoparticles–porous silicon	Sol–gel	3.37	2 ppm	2 min/20 min	25 °C	[48]
NiO/WO_3_ nanocomposites	Chemical method	4.8	30 ppm	2.5 s/1.1 s	25 °C	[49]
WO_3_	PLD	17	200 ppm	-	300 °C	[50]
WO_3_-Au	PLD + Sputtering	69	200 ppm	-	300 °C	[50]
SnO_2_-doped WO_3_	PLD	39.5	20 ppm	6 s/13 s	150 °C	[51]
As-deposited WO_3−x_	PLD	5	5 ppm	20 s/80 s	75 °C	This work
As-deposited WO_3−x_	PLD	93	20 ppm	20 s/80 s	75 °C	This work

## Data Availability

Data will be provided upon request.
